# Supplementary material for: A fault-intrusion-tolerant system and deadline-aware algorithm for scheduling scientific workflow in the cloud
Source: PeerJ Comput Sci. 2021 Nov 2;7:e747. doi: 10.7717/peerj-cs.747 (PMC8576554; doi:10.7717/peerj-cs.747)
Supplement: Supplemental Information 6 [file peerj-cs-07-747-s006.zip › Myproject1.1/docs/org/workflowsim/Task.html]

Task


JavaScript is disabled on your browser.


- Overview
- Package
- Class
- Use
- Tree
- Deprecated
- Index
- Help

- Prev Class
- Next Class

- Frames
- No Frames

- All Classes

- Summary:
- Nested |
- Field |
- Constr |
- Method

- Detail:
- Field |
- Constr |
- Method


org.workflowsim

## Class Task

- java.lang.Object
- - org.cloudbus.cloudsim.Cloudlet
  - - org.workflowsim.Task

- Direct Known Subclasses:
  :   Job

  ---

    

  ```
  public class Task
  extends Cloudlet
  ```

  Task is an extention to Cloudlet in CloudSim. It supports the implementation
  of dependencies between tasks, which includes a list of parent tasks and a
  list of child tasks that it has. In WorkflowSim, the Workflow Engine assure
  that a task is released to the scheduler (ready to run) when all of its
  parent tasks have completed successfully

  Since:
  :   WorkflowSim Toolkit 1.0

- - ### Field Summary

    - ### Fields inherited from class org.cloudbus.cloudsim.Cloudlet

      `accumulatedBwCost, CANCELED, costPerBw, CREATED, FAILED, FAILED_RESOURCE_UNAVAILABLE, INEXEC, PAUSED, QUEUED, READY, RESUMED, SUCCESS, vmId`
  - ### Constructor Summary

    Constructors

    Constructor and Description

    `Task(int taskId,
    long taskLength)`

    Allocates a new Task object.
  - ### Method Summary

    Methods

    | Modifier and Type | Method and Description |
    | --- | --- |
    | `void` | `addChild(Task task)` Adds a task to existing child list |
    | `void` | `addChildList(java.util.List list)` Adds the list to existing child list |
    | `void` | `addFile(File file)` Adds a file to existing file list |
    | `void` | `addParent(Task task)` Adds a task to existing parent list |
    | `void` | `addParentList(java.util.List list)` Adds the list to existing parent list |
    | `java.util.List<Task>` | `getChildList()` Gets the child list of the task |
    | `int` | `getDepth()` Gets the depth of the task |
    | `java.util.List` | `getFileList()` Gets the list of the files |
    | `double` | `getImpact()` Gets the impact of the task |
    | `java.util.List<Task>` | `getParentList()` Gets the list of the parent tasks |
    | `int` | `getPriority()` Gets the priority of the task |
    | `java.lang.String` | `getType()` Gets the type of the task |
    | `void` | `setChildList(java.util.List list)` Sets the child list of the task |
    | `void` | `setDepth(int depth)` Sets the depth of the task |
    | `void` | `setFileList(java.util.List<File> list)` Sets a file list |
    | `void` | `setImpact(double impact)` Sets the impact factor |
    | `void` | `setParentList(java.util.List list)` Sets the parent list of the task |
    | `void` | `setPriority(int priority)` Sets the priority of the task |
    | `void` | `setType(java.lang.String type)` Sets the type of the task |

    - ### Methods inherited from class org.cloudbus.cloudsim.Cloudlet

      `addRequiredFile, deleteRequiredFile, getActualCPUTime, getActualCPUTime, getAllResourceId, getAllResourceName, getClassType, getCloudletFileSize, getCloudletFinishedSoFar, getCloudletFinishedSoFar, getCloudletHistory, getCloudletId, getCloudletLength, getCloudletOutputSize, getCloudletStatus, getCloudletStatusString, getCloudletTotalLength, getCostPerSec, getCostPerSec, getExecStartTime, getFinishTime, getNetServiceLevel, getNumberOfPes, getProcessingCost, getRequiredFiles, getReservationId, getResourceById, getResourceId, getResourceName, getStatus, getStatusString, getSubmissionTime, getSubmissionTime, getUserId, getUtilizationModelBw, getUtilizationModelCpu, getUtilizationModelRam, getUtilizationOfBw, getUtilizationOfCpu, getUtilizationOfRam, getVmId, getWaitingTime, getWallClockTime, getWallClockTime, hasReserved, isFinished, requiresFiles, setClassType, setCloudletFinishedSoFar, setCloudletLength, setCloudletStatus, setExecParam, setExecStartTime, setNetServiceLevel, setNumberOfPes, setRequiredFiles, setReservationId, setResourceParameter, setResourceParameter, setSubmissionTime, setUserId, setUtilizationModelBw, setUtilizationModelCpu, setUtilizationModelRam, setVmId, write`
    - ### Methods inherited from class java.lang.Object

      `clone, equals, finalize, getClass, hashCode, notify, notifyAll, toString, wait, wait, wait`

- - ### Constructor Detail


    - #### Task

      ```
      public Task(int taskId,
          long taskLength)
      ```

      Allocates a new Task object. The task length should be greater than or
      equal to 1.

      Parameters:
      :   `taskId` - the unique ID of this Task
      :   `taskLength` - the length or size (in MI) of this task to be executed
          in a PowerDatacenter
  - ### Method Detail


    - #### setType

      ```
      public void setType(java.lang.String type)
      ```

      Sets the type of the task

      Parameters:
      :   `type` - the type


    - #### getType

      ```
      public java.lang.String getType()
      ```

      Gets the type of the task

      Returns:
      :   the type of the task


    - #### setPriority

      ```
      public void setPriority(int priority)
      ```

      Sets the priority of the task

      Parameters:
      :   `priority` - the priority


    - #### setDepth

      ```
      public void setDepth(int depth)
      ```

      Sets the depth of the task

      Parameters:
      :   `depth` - the depth


    - #### getPriority

      ```
      public int getPriority()
      ```

      Gets the priority of the task

      Returns:
      :   the priority of the task


    - #### getDepth

      ```
      public int getDepth()
      ```

      Gets the depth of the task

      Returns:
      :   the depth of the task


    - #### getChildList

      ```
      public java.util.List<Task> getChildList()
      ```

      Gets the child list of the task

      Returns:
      :   the list of the children


    - #### setChildList

      ```
      public void setChildList(java.util.List list)
      ```

      Sets the child list of the task

      Parameters:
      :   `list,` - child list of the task


    - #### setParentList

      ```
      public void setParentList(java.util.List list)
      ```

      Sets the parent list of the task

      Parameters:
      :   `list,` - parent list of the task


    - #### addChildList

      ```
      public void addChildList(java.util.List list)
      ```

      Adds the list to existing child list

      Parameters:
      :   `list,` - the child list to be added


    - #### addParentList

      ```
      public void addParentList(java.util.List list)
      ```

      Adds the list to existing parent list

      Parameters:
      :   `list,` - the parent list to be added


    - #### getParentList

      ```
      public java.util.List<Task> getParentList()
      ```

      Gets the list of the parent tasks

      Returns:
      :   the list of the parents


    - #### addChild

      ```
      public void addChild(Task task)
      ```

      Adds a task to existing child list

      Parameters:
      :   `task,` - the child task to be added


    - #### addParent

      ```
      public void addParent(Task task)
      ```

      Adds a task to existing parent list

      Parameters:
      :   `task,` - the parent task to be added


    - #### getFileList

      ```
      public java.util.List getFileList()
      ```

      Gets the list of the files

      Returns:
      :   the list of files


    - #### addFile

      ```
      public void addFile(File file)
      ```

      Adds a file to existing file list

      Parameters:
      :   `file,` - the file to be added


    - #### setFileList

      ```
      public void setFileList(java.util.List<File> list)
      ```

      Sets a file list

      Parameters:
      :   `list,` - the file list


    - #### setImpact

      ```
      public void setImpact(double impact)
      ```

      Sets the impact factor

      Parameters:
      :   `impact,` - the impact factor


    - #### getImpact

      ```
      public double getImpact()
      ```

      Gets the impact of the task

      Returns:
      :   the impact of the task


- Overview
- Package
- Class
- Use
- Tree
- Deprecated
- Index
- Help

- Prev Class
- Next Class

- Frames
- No Frames

- All Classes

- Summary:
- Nested |
- Field |
- Constr |
- Method

- Detail:
- Field |
- Constr |
- Method
